# Modulation of cAMP/cGMP signaling as prevention of congenital heart defects in Pde2A deficient embryos: a matter of oxidative stress

**DOI:** 10.1038/s41419-024-06549-1

**Published:** 2024-02-23

**Authors:** Silvia Cardarelli, Martina Biglietto, Tiziana Orsini, Valentina Fustaino, Lucia Monaco, Ana Gabriela de Oliveira do Rêgo, Francesca Liccardo, Silvia Masciarelli, Francesco Fazi, Fabio Naro, Luciana De Angelis, Manuela Pellegrini

**Affiliations:** 1 Department of Anatomical, Histological, Forensic and Orthopaedic Sciences, Sapienza University of Rome, 00161 Rome, Italy; 2 Institute of Biochemistry and Cell Biology, IBBC-CNR, 00015 Monterotondo Scalo, Rome, Italy; 3 Department of Physiology and Pharmacology, Sapienza University of Rome, 00185 Rome, Italy

**Keywords:** Mechanisms of disease, Congenital heart defects

## Abstract

Phosphodiesterase 2A (Pde2A) is a dual-specific PDE that breaks down both cAMP and cGMP cyclic nucleotides. We recently highlighted a direct relationship between Pde2A impairment, a consequent increase of cAMP, and the appearance of mouse congenital heart defects (CHDs). Here we aimed to characterize the pathways involved in the development of CHDs and in their prevention by pharmacological approaches targeting cAMP and cGMP signaling. Transcriptome analysis revealed a modulation of more than 500 genes affecting biological processes involved in the immune system, cardiomyocyte development and contractility, angiogenesis, transcription, and oxidative stress in hearts from *Pde2A*^*−/−*^ embryos. Metoprolol and H89 pharmacological administration prevented heart dilatation and hypertabeculation in *Pde2A*^*−/−*^ embryos. Metoprolol was also able to partially impede heart septum defect and oxidative stress at tissue and molecular levels. Amelioration of cardiac defects was also observed by using the antioxidant NAC, indicating oxidative stress as one of the molecular mechanisms underpinning the CHDs. In addition, Sildenafil treatment recovered cardiac defects suggesting the requirement of cAMP/cGMP nucleotides balance for the correct heart development.

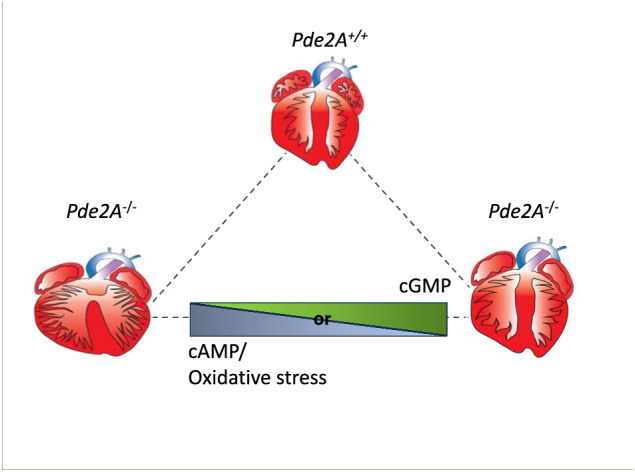

## Introduction

Phosphodiesterase 2A (Pde2A) hydrolyzes both cyclic nucleotides (cAMP and cGMP) and is activated by cGMP. Knockout mouse models for the different PDE isoforms have been generated (Supplementary Table S1) and up to date, the Pde2A knockout mouse is the only embryonically lethal, most likely due to an extremely reduced liver size, impairment of the hepatic niche, and elevated anemia [1, 2].

We recently reported that hearts of *Pde2A*^*−/−*^ embryos and hearts of embryos treated with a selective Pde2A inhibitor show ventricular and atrial septum defects, hypertrabeculation, heart dilatation, and non-compaction defects [3, 4].

We also demonstrated a direct relationship between Pde2A deficiency, the consequent increase in cAMP, and the occurrence of mouse congenital heart defects (CHDs). Indeed, cAMP has been shown to significantly transform fetal cardiomyocytes into cardiac pacemaker-like cells during cardiogenesis, increasing gap-junction markers [5, 6], promoting phosphorylation of targets such as L-type calcium channel via Protein Kinase A (PKA), and decreasing its concentration during heart development [7, 8].

On the contrary, an increase in local pools of cAMP-regulated by pharmacological Pde2A inhibition showed an anti-hypertrophic phenotype in adult mouse hearts [9].

Pde2A is localized at the plasma membrane, in the cytoplasm, and in the nucleus of neonatal murine cardiomyocytes [4, 10]. The Pde2A2 isoform was also described to be localized in the mitochondrial matrix and its inhibition to stimulate oxidative phosphorylation [11]. Recently, cAMP/PKA signaling domains localized at mitochondrial membranes and regulated by Pde2A2 were described to be involved in mitochondrial morphological change and apoptosis [12]. Because of this specific localization and function, we might hypothesize a contribution of Pde2A in reactive oxygen species (ROS) production. Indeed, ROS have traditionally been regarded as byproducts of aerobic metabolism, with mitochondrial respiration being the major intracellular source of accidental ROS production [13].

The stimulation of β-1 adrenergic receptor (β1-AdR) is the primary source of cAMP in the heart through adenylyl cyclase (AC) activation [14]. Several uncontrolled and controlled clinical studies provided the first evidence that Metoprolol, a selective β1-adrenoceptor blocking agent (β-blocker), had beneficial clinical and hemodynamic effects in patients with heart failure due to idiopathic dilated cardiomyopathy (DCM) [15–19]. Metoprolol was able to extend survival in DCM mice and prevent cardiac remodeling and dysfunction [20, 21].

The cAMP target PKA plays several functions in the normal heart such as contraction, metabolism, and gene transcription regulation. However, increased PKA activity and protein levels were described to aggravate heart failure [22–24]. PKA activators induced ROS and consequent electrical disturbance in heart failure, whereas PKA inhibition with H89 prevented it in humans [25]. H89 administration resulted also in the attenuation of DCM and the prevention of heart failure [24]. Moreover, it has been demonstrated to decrease cell death in heart disease and to improve cardiac function after myocardial infarction in mice [26].

It is also well known that cGMP plays a protective role in cardiac physiology and sildenafil, a selective inhibitor of PDE5A, acts in heart remodeling by increasing cGMP levels [27, 28].

Here, investigating the transcriptome of Pde2A deficient embryonic hearts by RNA sequencing (RNA-seq) analysis, we found a significant modulation of more than 500 genes associated with biological processes such as immune response, cardiomyocyte development, and contractility, angiogenesis, control of gene transcription and oxidative stress. Testing pharmacological treatments that blunt cAMP/PKA signaling, we observed that Metoprolol and H89 can robustly prevent CHDs in Pde2A deficient embryos similarly to N-acetyl-cysteine (NAC) antioxidant administration. Moreover, among the pathways severely up-regulated, we found that Metoprolol treatment reestablishes the expression levels of oxidative stress-related genes and NAC reduces in vivo heart oxidative stress. Finally, a pharmacological treatment affecting cGMP signaling was found to ameliorate cardiac defects to a similar extent.

## Results

### RNA-seq analysis reveals alteration of pathways involved in immune system, angiogenesis, oxidative stress, gene transcription and cardiogenesis

We previously showed downregulation of critical genes involved in cardiac development in *Pde2A* knockout embryos when compared to wild types [3]. To better define the global transcriptional profile of the two genotypes, RNA-seq was performed on isolated hearts from *Pde2A*^*+/+*^ and *Pde2A*^*−/−*^ embryos at E14.5.

**Fig. 1:**
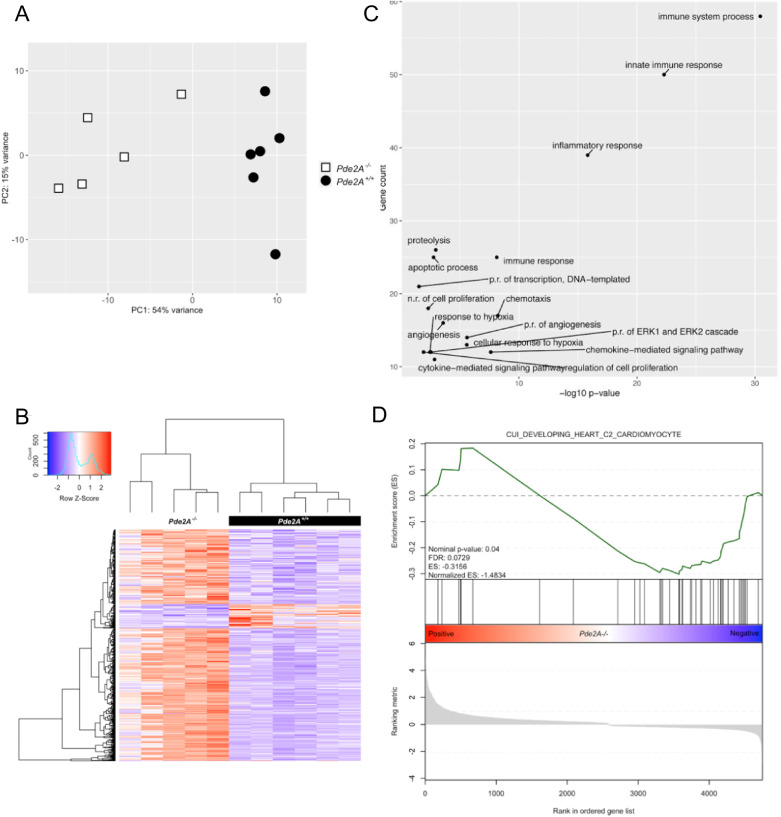
RNA-seq data analysis reveals gene expression dysregulation in hearts of E14.5 *Pde2A*^*−/−*^. **A** Plots of samples on first (PC1) and second (PC2) principal components of RNA-seq data. The PC percentage of variance is reported. **B** Heatmap representation of 515 differentially expressed genes considering a log2-fold change >+1 and <−1 and *p*-adjusted < 0.05. **C** Gene ontology biological processes with a significant enrichment (*p*-value < 0.05 and gene count > 10) in the list of *Pde2A*^*−/−*^ differentially expressed genes (*n* = 515). **D** Gene set enrichment analysis (GSEA) of *Pde2A*^*−/−*^ differentially expressed genes (*n* = 515) by the cardiomyocyte gene set of the Molecular Signatures Database (MSigDB); *n* = 6 *Pde2A*^*+/+*^ and *n* = 5 *Pde2A*^*−/−*^ hearts.

Principal component analysis (PCA) indicated that *Pde2A*^*−/−*^ was consistently different from *Pde2A*^*+/+*^, with the first component showing more than 50% of the global variance (Fig. 1A). The extensive alteration in the expression profile between *Pde2A*^*−/−*^ and *Pde2A*^*+/+*^ was also displayed by the heatmap (Fig. 1B): a stringent analysis (fold change ≥ 2; *p*-value < 0.05) found 55 downregulated genes and 460 upregulated genes in the *Pde2A*^*−/−*^ with respect to *Pde2A*^*+/+*^embryonic hearts (Supplementary Table S2). A subsequent gene ontology (GO) analysis using the DAVID functional enrichment on biological processes revealed that upregulated genes were involved in pathways such as immune system and inflammatory response, chemokine-mediated signaling, angiogenesis, cellular response to hypoxia, and positive regulation of ERK1 and ERK2 cascades (Fig. 1C). The differentially expressed genes were investigated using the gene set enrichment analysis (GSEA) and compared to gene sets from Molecular Signatures Database (MSigDB) to characterized different cellular populations enriched in *Pde2A*^*−/−*^ hearts. In line with our previous data, the cardiomyocyte lineage negatively correlated with *Pde2A*^*−/−*^ (Fig. 1D), whereas the other lineages such as endothelial, fibroblast, smooth muscle cells, macrophages, are strongly and positively correlated with *Pde2A*^*−/−*^ (Supplementary Fig. S1).

**Fig. 2:**
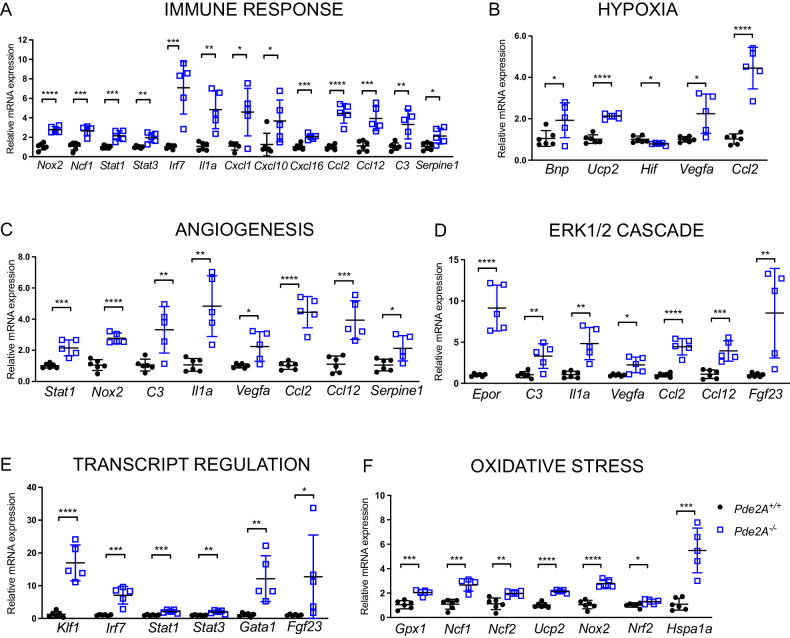
Real-time PCR confirms up-regulation of selected genes from biological processes. Histograms of relative mRNA expression of genes up-regulated in RNA-seq analysis and enriched in biological processes for immune response (**A**), hypoxia (**B**), angiogenesis (**C**), ERK1/2 cascade (**D**), trancription regulation (**E**), oxidative stress (**F**). Each dot represents an embryo; *n* = 5 *Pde2A*^*+/+*^ and *n* = 5 *Pde2A*^*−/−*^ hearts; unpaired Student’s *t*-test *P ≤ 0.05, **P ≤ 0.01, ***P ≤ 0.001, ****P ≤ 0.0001.

Some genes, associated with enriched pathways found in DAVID analysis, were validated by RT-qPCR (Fig. 2). We selected genes linked to the immune system/inflammation (Fig. 2A), genes involved in angiogenesis and hypoxia (Fig. 2B, C), genes involved in survival, proliferation, and transcription (Fig. 2D, E). Some genes, such as *Ccl2, Vegfa*, *Il1a*, and *Fgf23*, were robustly upregulated and expressed in different biological processes. Real-time PCR fully confirmed the RNA-seq analysis. A deeper investigation of RNA-seq results highlighted that also several genes involved in oxidative stress were altered in *Pde2A*^*−/−*^ embryonic hearts (Supplementary Table S2). Real-time PCR analysis confirmed that most of them were upregulated in knockout hearts at E14.5 (Fig. 2F). These results indicated that in *Pde2A*^*−/−*^ hearts there is a high level of inflammation, oxidative stress, angiogenesis, and a reduction of cardiomyocyte differentiation, at least at the transcriptional level.

### Metoprolol treatment prevents CHDs of *Pde2A*^*−/−*^ embryos

Pde2A can hydrolyze both cAMP and cGMP and, moreover, its catalytic activity rises when cGMP binds allosterically to the Pde2A GAF-B domain.

We measured the cyclic nucleotide levels in embryonic hearts from *Pde2A*^*+/+*^ and *Pde2A*^*−/−*^ and we observed that the cAMP level is almost doubled in knockout compared to wild-type hearts at E14.5 (Supplementary Fig. S2A), as previously published [3], whereas negligible differences were found in cGMP content (Supplementary Fig. S2B).

In addition, referring to the KEGG PATHWAY database, the RNA-seq data were queried in order to compare the expression of genes involved in cAMP (KEGG ID: mmu04024) and cGMP (KEGG ID: mmu04022) pathways in the heart of *Pde2A*^*−/−*^ and *Pde2A*^*+/+*^ embryos (Supplementary Fig. S3A, B). We found a total of 63 genes for the cAMP pathway and 50 genes for the cGMP pathway with a significative up- or down-regulation in *Pde2A*^*−/−*^. Among these, 24 genes were common to both cyclic nucleotides (Supplementary Fig. S3C). Specific highly modulated cAMP and cGMP-dependent genes were 15 and 5, respectively (Supplementary Fig. S3C).

**Fig. 3:**
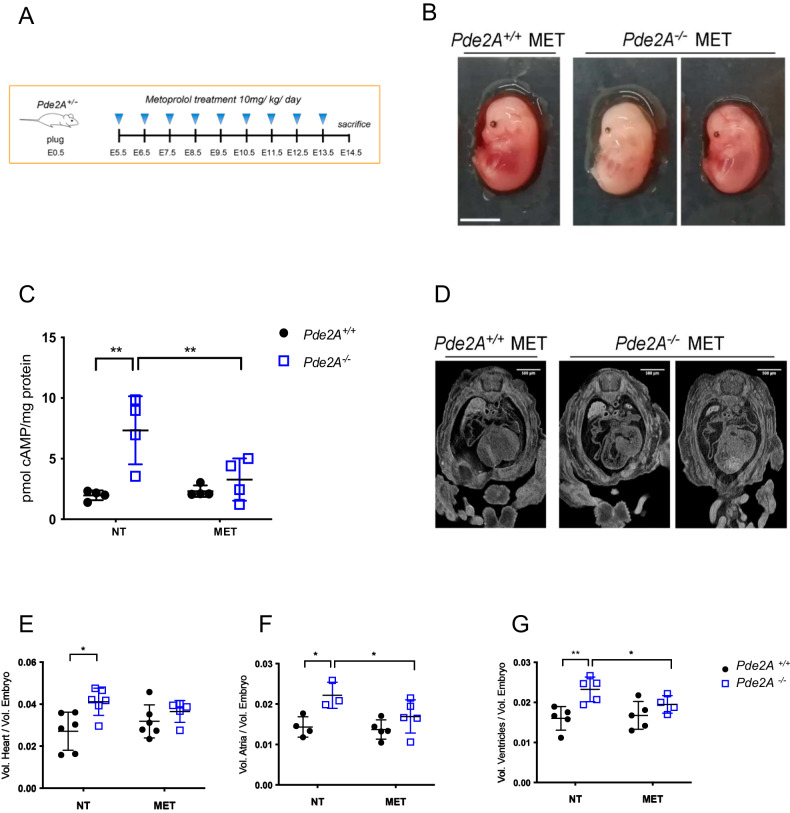
Micro-CT examination shows heart defect recovery after Metoprolol treatment. **A** Scheme of Metoprolol (MET) treatment. **B** Picture of *Pde2A*^*+/+*^ and *Pde2A*^*−/−*^ embryos at E14.5 treated with Metoprolol. Scale bar = 0.5 cm. **C** cAMP level in *Pde2A*^*+/+*^ and *Pde2A*^*−/−*^ in hearts of Metoprolol treated. *n* = 4 for each condition and genotype; ANOVA two-way was used to compare *Pde2A*^*−/−*^ versus the relative *Pde2A*^*+/+*^ in both conditions and *Pde2A*^*−/−*^ not treated versus *Pde2A*^*−/−*^ Metoprolol samples; **P ≤ 0.01. **D** Micro-CT picture of the hearts in Metoprolol-treated *Pde2A*^*+/+*^ and *Pde2A*^*−/−*^ embryos. **E**–**G** Ratio between total heart (**E**), atrial (**F**), and ventricular (**G**) volumes relative to embryo volumes obtained by micro-CT analyses of Metoprolol treated or not treated (NT) *Pde2A*^*+/+*^ and *Pde2A*^*−/−*^ embryos. At least *n* = 4 for each genotype/treatment. ANOVA two-way was used to compare *Pde2A*^*−/−*^ versus the relative *Pde2A*^*+/+*^ in both conditions and *Pde2A*^*−/−*^ not treated versus *Pde2A*^*−/−*^ Metoprolol samples; *P ≤ 0.05, **P ≤ 0.01.

Initially, we focused on pharmacological treatments that directly impact cAMP signaling to test whether they could rescue cardiac malformations in *Pde2A*^*−/−*^ embryos. Metoprolol, a selective antagonist of β1-adrenergic receptor and consequently of cAMP synthesis, was administered after implantation to pregnant female mice every day, starting at E5.5 from the plug until E13.5, the day before sacrifice (Fig. 3A).

**Fig. 4:**
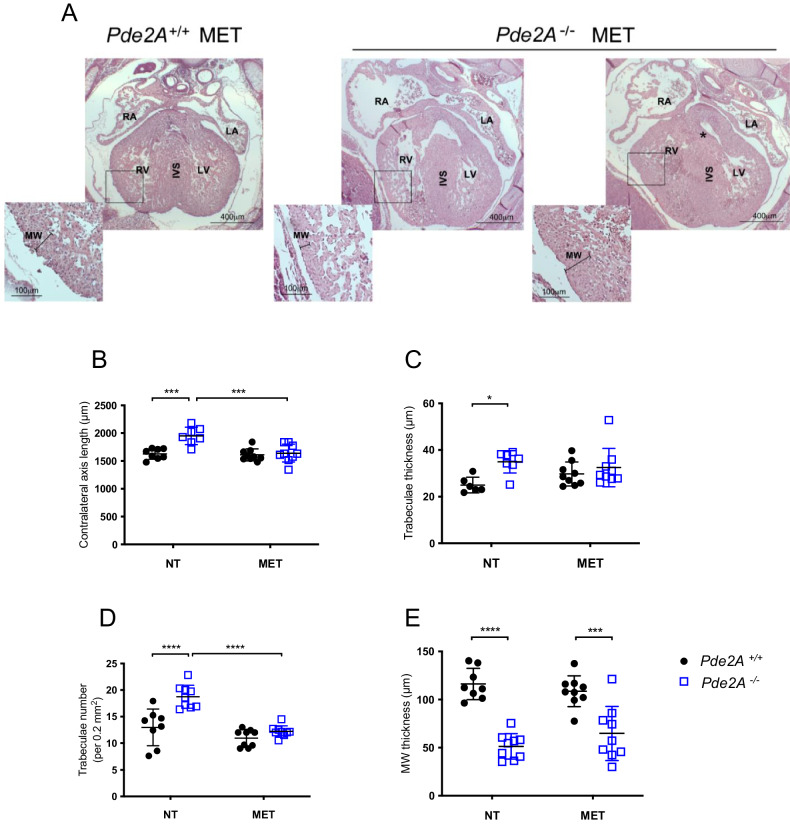
Histological analyses show heart defects recovery, except in the myocardial wall, after Metoprolol treatment. **A** Haematoxylin and Eosin staining of transversal sections of Metoprolol treated (MET) embryos, the heart is shown. Left and right ventricles (LV, RV), atria (LA, RA), and interventricular septum (IVS) are indicated. Insets show magnification of trabeculae and myocardial wall (MW). **B**–**E** Graphs of contralateral axis (**B**), trabeculae thickness (**C**), trabeculae number (**D**), and myocardial wall (**E**) analyses in treated and not treated (NT) samples. *n* = 9 *Pde2A*^*+/+*^ and *n* = 11 *Pde2A*^*−/−*^ not treated embryos and *n* = 4 *Pde2A*^*+/+*^ and *n* = 3 *Pde2A*^*−/−*^ Metoprolol treated embryos; ANOVA two-way was used to compare *Pde2A*^*−/−*^ versus the relative *Pde2A*^*+/+*^ in both conditions and *Pde2A*^*−/−*^ not treated versus *Pde2A*^*−/−*^ Metoprolol samples. *P ≤ 0.05, ***P ≤ 0.001, ****P ≤ 0.0001.

Morphological examination of Metoprolol-treated E14.5 *Pde2A*^*−/−*^ embryos revealed anemia, hemorrhages, and reduced liver size, as shown in *Pde2A*^*−/−*^ untreated embryos [3] (Fig. 3B and Supplementary Fig. S4A, B). The level of cAMP was restored and became similar in *Pde2A*^*+/+*^ and *Pde2A*^*−/−*^ hearts after Metoprolol treatment, validating the treatment efficacy (Fig. 3C). In addition, micro-CT analysis revealed similar parameters between *Pde2A*^*−/−*^ embryonic hearts treated with Metoprolol and *Pde2A*^*+/+*^ hearts; total heart volume, atrial volume, and ventricular volume normalized to embryos volume did not show difference in wild-type versus knockout treated embryos contrary to wild-type versus knockout untreated embryos (Fig. 3D–G). Moreover, half of the *Pde2A*^*−/−*^ treated embryos had no interventricular septum defects (Fig. 3D and Supplementary Video S1–S3). Morphological and morphometric analyses of the hearts were then performed on hematoxylin and eosin-stained sections of paraffin-embedded E14.5 embryos. Contrary to untreated embryos, no differences in the contralateral axis between *Pde2A*^*+/+*^ and *Pde2A*^*−/−*^ treated hearts were observed (Fig. 4A, B), confirming the ability of Metoprolol to prevent cardiac enlargement. Similarly, the hypertrabeculation of *Pde2A*^*−/−*^ hearts was improved by Metoprolol administration, and wild-type and knockout embryos showed similar thickness and number of cardiac trabeculae, respectively (Fig. 4A, C, D). Furthermore, immunofluorescence for endomucin, which marks the endocardium, revealed trabecular network reconstitution after Metoprolol treatment in *Pde2A*^−/−^ embryos (Supplementary Fig. S5). However, a significant thinning of the ventricular myocardium wall persisted in *Pde2A*^*−/−*^ heart after Metoprolol treatment, even though a trend of thickness increase was observed (Fig. 4A–E). Hematoxylin and Eosin staining confirmed that half of treated *Pde2A*^−/−^ hearts did not show ventricular septum defects (Fig. 4A). These results suggest that Metoprolol, reestablishing the cAMP levels, can partially revert *Pde2A*^*−/−*^ cardiac defects, probably acting on some of the pathways specifically controlled by the Pde2A/cAMP system.

### Metoprolol treatment restores oxidative stress response and the antioxidant NAC ameliorates cardiac defects in hearts of *Pde2A*^*−/−*^ embryos

**Fig. 5:**
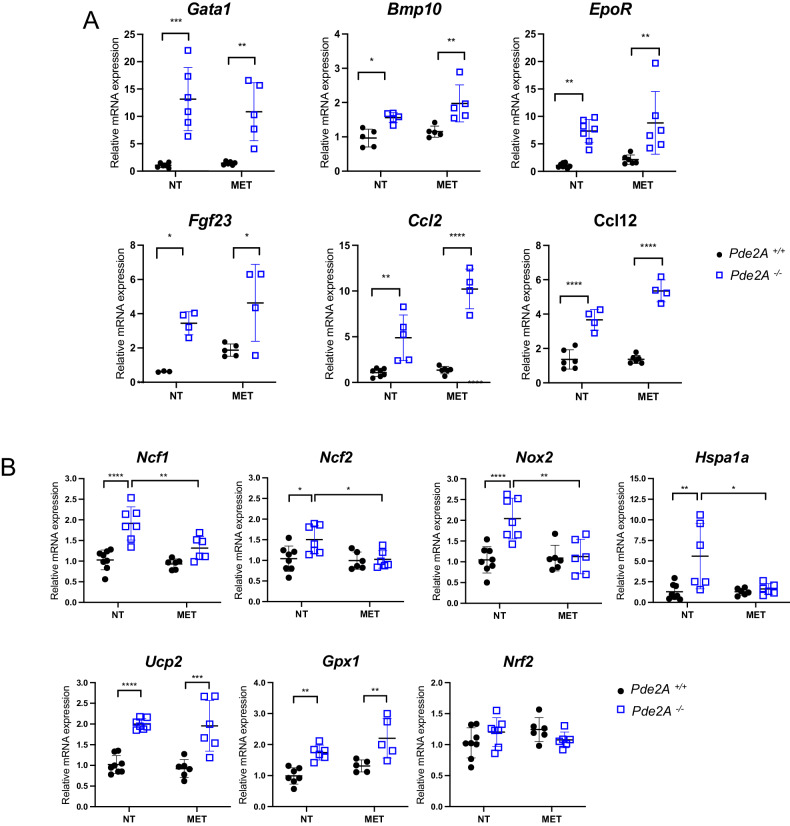
Prevention of oxidative stress gene up-regulation by Metoprolol treatment. **A** Histograms of relative mRNA expression of most up-regulated genes involved in different biological processes evaluated by RT-qPCR. **B** Histograms of relative mRNA expression of critical oxidative stress genes evaluated by RT-qPCR. *n* = 9 *Pde2A*^*+/+*^ and *n* = 11 *Pde2A*^*−/−*^ not treated (NT) embryos and *n* = 4 *Pde2A*^*+/+*^ and *n* = 3 *Pde2A*^*−/−*^ Metoprolol (MET) treated embryos; ANOVA two-way was used to compare *Pde2A*^*−/−*^ versus the relative *Pde2A*^*+/+*^ in both conditions and *Pde2A*^*−/−*^ not treated versus *Pde2A*^*−/−*^ Metoprolol samples. *P ≤ 0.05, **P ≤ 0.01, ***P ≤ 0.001, ****P ≤ 0.0001.

We then evaluated the expression level of genes that resulted more upregulated in *Pde2A* knockout hearts by RNA-seq analysis and common to different pathways, in Metoprolol treated and untreated embryos. We observed that except for Fgf23, Metoprolol treatment did not affect the upregulation of Gata1 and Bmp10 found in *Pde2A*^*−/−*^ as well as the expression increase of Epor, Ccl2, and Ccl12 genes. (Fig. 5A). Vice versa, the expression of several genes involved in oxidative stress, was reverted by Metoprolol treatment (Fig. 5B). These results suggest that oxidative stress is counteracted by reestablishing the functional cAMP/PKA signaling.

**Fig. 6:**
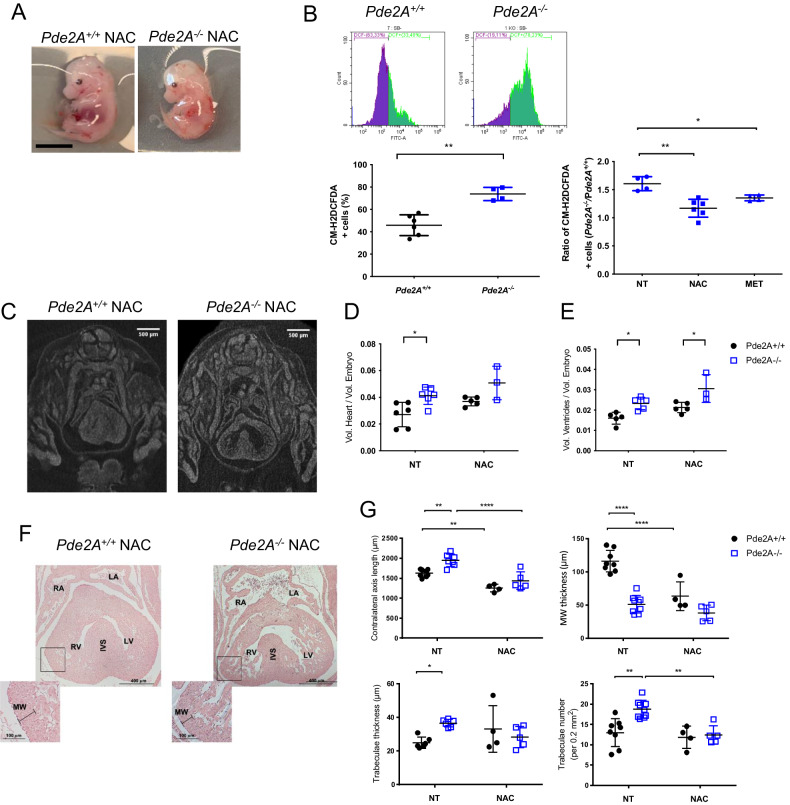
Oxidative stress and specific CHDs prevention by NAC administration. **A** Picture of *Pde2A*^*+/+*^ and *Pde2A*^*−/−*^ embryos at E14.5 treated with NAC. Scale bar = 0.5 cm. **B** Flow cytometry analysis of ROS level in cardiac cells isolated from *Pde2A*^*+/+*^ and *Pde2A*^*−/−*^ heart embryos in the presence or absence of NAC or Metoprolol (MET). At least *n* = 3 for each genotype and treatment. Student *t*-test was used to compare *Pde2A*^*−/−*^ versus *Pde2A*^*+/+*^ CM-H2DCFD+ cells and ANOVA one-way to compare the *Pde2A*^*−/−*^/*Pde2A*^*+/+*^ ratio of NAC or MET treatment versus untreated samples. *P ≤ 0.05, **P ≤ 0.01. **C** Micro-CT picture of the hearts in NAC-treated *Pde2A*^*+/+*^ and *Pde2A*^*−/−*^ embryos. Scale bar = 0.5 cm. **D** and **E** Ratio between total heart (**D**), ventricular (**E**) volumes relative to embryo volumes obtained by micro-CT analyses of NAC treated or not treated (NT) *Pde2A*^*+/+*^ and *PDE2A*^*−/−*^ embryos. At least n = 3 for each genotype /treatment. **F** Haematoxylin and Eosin staining of transversal sections of NAC treated or NT embryos, the heart is shown. Scale bar = 400 µm. **G** Graphs of contralateral axis, trabeculae thickness, trabeculae number, and myocardial analyses in treated and not-treated samples. At least *n* = 6 *Pde2A*^*+/+*^ and *n* = 6 *Pde2A*^*−/−*^ NT embryos and *n* = 4 *Pde2A*^*+/+*^ and *n* = 5 *Pde2A*^*−/−*^ NAC treated embryos. ANOVA two-way was used to compare *Pde2A*^*−/−*^ versus the relative *Pde2A*^*+/+*^ in both conditions and *Pde2A*^*−/−*^ not treated versus *Pde2A*^*−/−*^ NAC samples. *P ≤ 0.05, **P ≤ 0.01, ****P ≤ 0.0001.

Based on previous results, we checked whether these alterations could be rescued by the antioxidant NAC treatments (Fig. 6). Phenotypically the NAC-treated mutant embryos appeared like the *Pde2A*^*−/−*^ embryos (Fig. 6A). The CM-H2DCFDA staining revealed that ROS positive cells were augmented in *Pde2A*^*−/−*^ isolated cardiac cells, compared to *Pde2A*^*+/+*^ cells confirming an increase of oxidative stress in the absence of Pde2A (Fig. 6B). NAC administration significantly counteracted ROS production in *Pde2A*^*−/−*^ cardiac samples (Fig. 6B). Metoprolol treatment was also evaluated for ROS production and a slight but significant reduction was observed (Fig. 6B).

Micro-CT analysis did not show major rescue in *Pde2A*^*−/−*^ embryos treated with NAC, except for total heart volume (Fig. 6C–E and Supplementary Videos S4, S5). However, histological analysis revealed improvement in parameters such as contralateral axis and trabecular number and thickness (Fig. 6F–G). To be noted NAC appeared to decrease some of these values also in *Pde2A*^*+/+*^embryos, suggesting an effect on NAC in the basal homeostasis of cardiac oxidative stress during development.

Overall, these results indicate that antioxidants can be a valuable treatment for CHDs caused by cAMP unbalance.

### H89 treatment partially rescues CHDs of *Pde2A*^*−/−*^ embryos

To validate the cause–effect of the increase of cAMP level in the onset of CHDs, we investigated downstream pathways by using a PKA inhibitor. Several PKA inhibitors have been developed, however, none of them is specific. We decided to use H89 that is more potent as a PKA inhibitor compared to its ability to inhibit various other protein kinases, such as S6K1, MSK1, PKA, ROCKII, PKBα, and MAPKAP-K1b [29, 30]. Moreover, H89 is largely employed in in vivo studies and, most importantly, already tested and well-tolerated during pregnancy [31]. Morphological examination of H89-treated *Pde2A*^*−/−*^ E14.5 embryos showed anemia, hemorrhages, and reduced liver size like not-treated embryos (Supplementary Fig. S6A). Micro-CT analysis revealed that H89 treatment was able to prevent the increase of total heart, atrial, and ventricular volumes observed in *Pde2A*^*−/−*^ respect to *Pde2A*^*+/+*^ embryos (Supplementary Fig. S6B, C). However, *Pde2A*^*−/−*^ H89-treated hearts maintained ventricular septum defects (Supplementary Fig. S6B and Video S6-S7).

Heart microscopic examination of hematoxylin and eosin-stained embryonic sections revealed that H89 was able to prevent the increase of the contralateral axis, the increase of trabecular number and thickness, but not the ventricular septum defect or the reduction of the myocardial wall in *Pde2A*^*−/−*^ (Supplementary Fig. S6D, E). These data strenghten the hypothesis that inhibition of the cAMP/PKA system, although partially, can rescue CHDs of *Pde2A*^*−/−*^ embryos.

### Sildenafil treatment prevents CHDs of *Pde2A*^*−/−*^ embryos

We next investigated whether the pharmacological treatment that directly impacts cGMP signaling could also prevent cardiac malformations in *Pde2A*^*−/−*^ embryos. Pharmacological treatment of pregnant *Pde2A*^*+/−*^ females with Sildenafil, a drug preserving cGMP levels by inhibiting Pde5A, was performed.

**Fig. 7:**
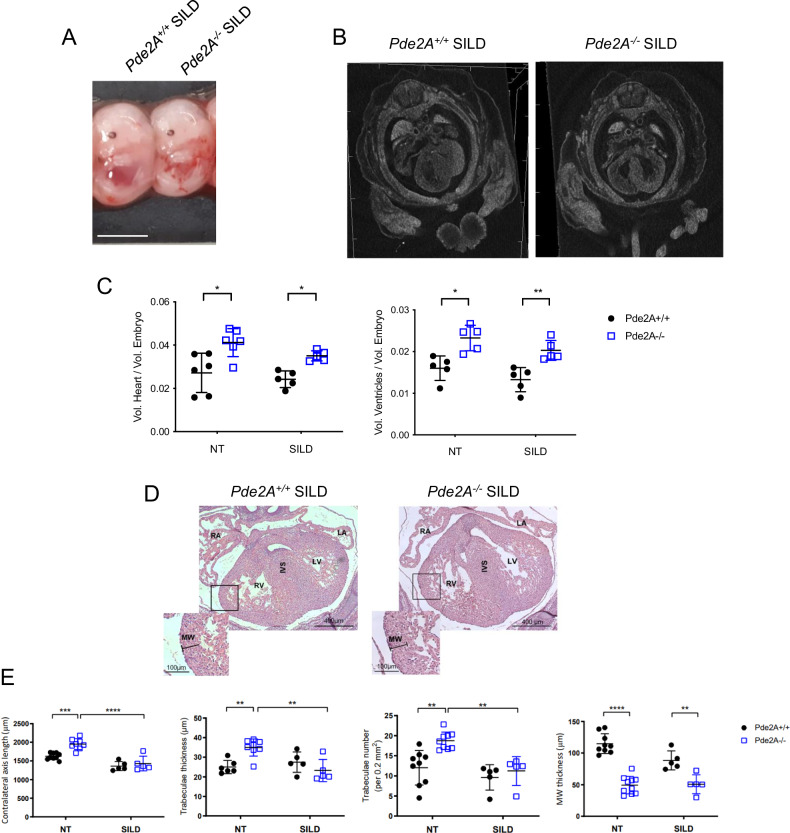
Histological examination shows heart defects recovery after Sildenafil treatment. **A** Picture of Sildenafil (SILD) treated *Pde2A*^*+/+*^ and *Pde2A*^*−/−*^ E14.5. embryos. Scale bar = 0.5 cm. **B** Micro-CT picture of the hearts in Sildenafil-treated *Pde2A*^*+/+*^ and *Pde2A*^*−/−*^ embryos. **C** Ratio between total hearts and ventricular volumes relative to embryo volumes obtained by micro-CT analyses of Sildenafil treated or not treated (NT) *Pde2A*^*+/+*^ and *Pde2A*^*−/−*^ embryos. At least *n* = 5 for each genotype/treatment. **D** Haematoxylin and Eosin staining of transversal sections of *Pde2A*^*+/+*^ and *Pde2A*^*−/−*^ Sildenafil treated embryos, the heart is shown. Left and right ventricles (LV, RV), atria (LA, RA), and interventricular septum (IVS) are indicated. Inset shows a magnification of trabeculae and myocardial wall (MW). **E** Graphs of contralateral axis, trabeculae thickness and trabeculae number, and myocardial wall analyses in treated and not treated samples. At least *n* = 5 for each condition/genotype. ANOVA two-way was used to compare *Pde2A*^*−/−*^ versus the relative *Pde2A*^*+/+*^ in both conditions and *Pde2A*^*−/−*^ not treated versus *Pde2A*^*−/−*^ Sildenafil samples. *P ≤ 0.05, **P ≤ 0.01, ***P ≤ 0.001 ****P ≤ 0.0001.

Macroscopic examination revealed no recovery of gross phenotypes of *Pde2A*^*−/−*^ embryos (Fig. 7A). On the contrary, micro-CT (Fig. 7B and Supplementary video S8, S9) and histological analyses (Fig. 7D, E) showed recovery of heart defects in terms of contralateral axis, trabecular number and thickness and ventricular septum defects (Fig. 7D, E), but not the heart volumes (Fig. 7C) or wall thickness (Fig. 7E). These data indicate that prevention of cGMP degradation leads to improvement of CHDs in *Pde2A*^*−/−*^ embryos.

## Discussion

In this study, RNA-seq was performed on isolated hearts from *Pde2A*^*+/+*^ and *Pde2A*^*−/−*^ at E14.5. Gene expression analysis revealed extensive alterations in the transcriptional profile of *Pde2A*^*−/−*^ hearts and 515 genes showed strong gene expression alteration. Notably, the transcriptome analysis revealed the deregulation of many pathways, including those involved in the immune response, angiogenesis, hypoxia, oxidative stress, and transcription factors involved in the ERK signaling cascade, by performing a stringent analysis with DAVID functional enrichment. All these pathways are important for correct cardiac development and their alterations have also been reported in various cardiovascular diseases [32].

Some of the genes found altered in *Pde2A*^*−/−*^ hearts, such as Epor, Ccl2, and Fgf23 are described to be involved in mitochondrial metabolism rather than in many other functions [33–35]. The binding of erythropoietin to its receptor, Epor, activates endothelial nitric oxide synthase (eNOS) which is involved in the regulation of mitochondrial biogenesis, turnover, and proliferation [33]. Ccl2 overexpression may be linked to alterations in mitochondrial dynamics that regulate anabolic and catabolic pathways, indicating a relationship between mitochondrial dysfunction, autophagy, and chronic disease [34]. High levels of Fgf23 are able to induce oxidative stress through the activation of NADPH oxidase complex [35].

Although it is widely known that inflammatory cytokines and oxidative, stress levels are elevated in numerous cardiovascular diseases [36, 37], only a few inconclusive studies have been published on the function of inflammation and oxidative stress in CHDs in neonates and adults [38–41].

Altogether, the RNA-seq results indicated an alteration of mitochondrial function in the knockout model for *Pde2A*. A deeper investigation confirmed that genes associated with oxidative stress such as Nox2, Ncf1, Ncf2, Nrf2, Ucp2, Hspa1a, and Gpx1 are significantly upregulated in *Pde2A*^*−/−*^ embryonic hearts.

Nox2 is the catalytic subunit of the mitochondrial NADPH oxidase (Nox) that, upon activation, binds various regulatory subunits such as neutrophil cytosolic factor 1 (Ncf1) and neutrophil cytosolic factor 2 (Ncf2) and plays an important role in the production of ROS [42]. Physiologically, the cells have developed various mechanisms to protect themselves from the accumulation of ROS. These mechanisms include the prevention of the accumulation of ROS through uncoupling protein 2 (Ucp2), the reduction of hydrogen peroxide mediated by the nuclear factor erythroid 2-related factor 2 (Nrf2), and ROS degradation via glutathione peroxidase 1 (Gpx1) [43, 44]. In case of stress, it is also important to maintain adequate protein homeostasis inside the cell. Indeed, the chaperon heat shock protein 1A (Hspa1a), during cellular stress, might activate two opposite mechanisms, the folding, and the degradation of proteins depending on its acetylation/deacetylation state [45].

We found increased levels of cAMP, but not cGMP in the heart of *Pde2A*^*−/−*^ embryos. However different local compartmentalization of cGMP in *Pde2A* deficient embryos could contribute to heart defects. In addition, the expression of genes specific to the cGMP pathway resulted to be highly altered in the RNA-seq analysis.

The Metoprolol and H89 used in this study are drugs of clinical use that act upstream and downstream of the cAMP cascade, respectively. Metoprolol was also administrated to pregnant women [46] and was reported to influence cGMP signaling [47].

Both drugs were not able to prevent the gross phenotypes of *Pde2A* knockout embryos such as liver size, anemia, and hemorrhages. However, E14.5 embryos from mothers who had Metoprolol treatment regained cardiac dimensions, and hypertrabeculation defect, and partially restored the interventricular septal defect. On the other hand, embryos harvested from mothers treated with H89 showed a recovery of cardiac dimension and hypertrabeculation, but not the interventricular septum. H89 clearly is effective in having an in vivo action, however, the exact identity of the targeted kinase(s) remains to be determined, this is because H89, as well as inhibiting PKA also inhibits various other kinases [29, 30].

Molecular analyses carried out on hearts of E14.5 *Pde2A*^*−/−*^ embryos showed that Metoprolol can reduce the level of cAMP and the expression of genes that induce oxidative stress (Nox2, Ncf1, Ncf2), whereas preserves the high expression of genes that protect from oxidative stress (Ucp2, Gpx1). It also decreased oxidative stress in the *Pde2A*^*−/−*^ cardiac cells, evaluated by CM-H2DFCDA staining, which specifically detects peroxidase formation. Vice versa, Metoprolol treatment does not affect the upregulation of genes implicated in the inflammatory response possibly because they trigger oxidative stress or because the left heart defects maintain an inflammatory state.

NAC is a safe and well-tolerated compound displaying antioxidant properties; it acts mainly by providing the intracellular cysteine necessary to increase glutathione levels. Our results indicate that NAC attenuated just some CHDs carried by *Pde2A*^*−/−*^ embryos, possibly because of the low drug bioavailability from the mother to the embryos. Dose increase, modification of drug administration procedures, or combination of NAC with other antioxidants and/or anti-inflammatory compounds may be more effective in preventing CHDs.

To investigate whether CHDs could be also due to cAMP/PKA independent effects such as altered local cGMP-dependent pathways or cyclic nucleotide-independent pathways, Sildenafil was used to increase cGMP levels and to reestablish the cAMP/cGMP balance in the *Pde2A*^*−/−*^ hearts. Indeed, Sildenafil ameliorates cardiac defects comparably to other pharmacological treatments suggesting the relevance of the cAMP/cGMP ratio for correct heart development.

Up to now, it is not yet clear to what extent the different types of cardiac cells are differentially affected in the heart of *Pde2A*^*−/−*^ versus *Pde2A*^*+/+*^ embryos and how the used drugs impact these cell populations. Future studies will address this topic by using flow cytometry and spatial digital transcriptomic analyses. To date, human CHDs related to the complete absence of PDE2A have not been found, probably because, as in the mouse model, homozygotes are embryonic lethal, or because other PDEs compensate for the lack of PDE2A.

Few recent reports were published describing patients with PDE2A mutations; in all these cases, the patients presented alterations related to the nervous system but no evaluation of cardiac performance with electrocardiograms, arrhythmias, and cardiac function was performed [48–50].

In conclusion, this study highlighted the involvement of Pde2A in new biological processes such as inflammation, angiogenesis, transcription, and oxidative stress and how pharmacological treatments, restoring the cyclic nucleotide balancing, can counteract congenital heart defects. Overall, the information obtained from our study will be useful to investigate human CHDs associated with impairment/imbalance of the cAMP/cGMP/PDE2A system and to investigate therapies suitable for their recovery.

## Methods

### Mouse breeding and in vivo treatments

*Pde2A*^*+/*−^ mice (B6; 129P2-Pde2A<tm1Dgen>/H; EM: 02366) were obtained from EMMA (UK). Timed mating was set up between *Pde2A*^*+*/−^ females and *Pde2A*^*+/*−^ males. Genotyping for Pde2A was performed as previously reported [3]. To test if pharmacological treatments, that affect directly or indirectly cAMP, can rescue cardiac malformations of *Pde2A*^*−/−*^ mice we chose two drugs that inhibit β1-adrenergic receptor signaling (Metoprolol) and PKA activity (H89), respectively. The drugs were administered every day starting at E5.5 from the plug until E13.5. Embryos were collected at E14.5.

Metoprolol (Sigma-Aldrich, MO, USA) was administered by gavage at 10 mg/kg dissolved in drinking water [20, 51]. H89 (H-89 dihydrochloride hydrate, Sigma-Aldrich, MO, USA) was administered intraperitoneal at 1 mg/kg [30, 52].

Sildenafil (Pfizer, NY, USA) was administered by gavage at Sildenafil 70 mg/kg/day dissolved in drinking water [53]. NAC (Sigma-Aldrich, MO, USA) was administered at 1 g/kg/day to *Pde2A*^*+/−*^ females in drinking water starting the first day of the plug and until day E14.5 [54]. Hearts of the resulting E14.5 embryos both males and females were isolated in Hanks balanced solution as previously described [3, 55] and processed for the following analyses. Only alive *Pde2A*^*+/+*^ and *Pde2A*^*−/−*^ were included in the study whereas abortions and *Pde2A*^*+/−*^ embryos were excluded. Plugged mothers and their embryos were randomly distributed in the not-treated or drug-treated groups. For each type of experiment, the minimal group size was estimated by the power analysis performed with G*Power 3.0.10 software using the following parameters: *α* = 0.05, 1−*β* = 0.85, and *d* = 1.

### Micro-CT imaging and volume measurements

*Imaging specimen preparation*: Embryos were fixed in 10% neutral buffered formalin (Bio-Optica, Italy) or 4% paraformaldehyde (PAF, Sigma-Aldrich, MO, USA) overnight at room temperature (RT) and embedded in paraffin following standard procedures.

*Micro-CT scanning*: Computed tomography images were acquired by a high-resolution 3D micro-CT imaging system (Skyscan 1172G Bruker, Kontich—Belgium), using a L7901-20 Microfocus X-ray Source (Hamamatsu). The acquisition of volumes was performed in 1.5 mL micro-tubes, with a camera pixel/size of 7.9 µm, camera binning 2 × 2, tube voltage peak of 39 kV, tube current of 240 µA, and exposure time of 450 ms. Reconstructions of tomographic datasets were performed using built-in NRecon Skyscan Software (Version:1.6.6.0; Bruker). The 3D volumes were analyzed using 3D Visualization Software CTvox v. 2.5 (Bruker). *Tissue segmentation*: Manual image-by-image segmentation aimed at the calculation of heart volume was applied, using Bruker micro-CT Analyzer Version 1.13 software. A histological atlas of mouse development [56] was used for guidance to accurately identify, demarcate, and segment each embryo (*n* = at least 3) in a specific volume of interest (VOI) for automated volume measurements [56, 57].

### Hematoxylin and eosin staining and immunofluorescence

Embryos at E14.5 were fixed in 10% neutral buffered formalin (Sigma-Aldrich) overnight at RT and included in paraffin (Sigma-Aldrich, MO, USA) following standard procedures. Serial paraffin sections of whole embryos (5 μm of thickness) were obtained, deparaffinized, and stained with hematoxylin and eosin (Sigma-Aldrich, MO, USA). Wall and trabeculae thickness, contralateral axis length, and trabeculae number were evaluated as previously described [3]. All histological analyses were performed in a blinded way by two different authors. The images were collected on a ZEISS axioskop 2 plus microscope mounting Axiocam 503 CCD camera and analyzed with the ImageJ Software (version 1.52t, NIH-Bethesda, MD, USA).

For immunofluorescence, 5 μm E14.5 paraffine sections underwent deparaffinization and microwave antigen retrieval in pH 6.0 sodium citrate with 0.05% Tween-20 solution (Sigma-Aldrich, MO, USA), followed by gradual chilling. Samples were permeabilized with 0.2% NP40 (Sigma-Aldrich, MO, USA) in phosphate-buffered saline (PBS) for 30 min and blocked with 1% bovine serum albumin (Sigma-Aldrich, MO, USA) in PBS for 1 h at room temperature. Sections were incubated overnight at 4 °C with anti-Endomucin antibody (Ab106100, Abcam, UK). After 3 × 5 min washes with PBS, sections were stained for 1 h at room temperature with anti-rat secondary antibody (Alexa Fluor 488; Thermo Fisher Scientific, MA, USA) followed by 2 min of Sudan Black (Sigma-Aldrich, MO, USA; 0.03g/10 ml 70% EtOH) to reduce the background and nuclei were counterstained with Hoechst (Sigma-Aldrich, MO, USA) for 5 min. Slides were mounted with Fluoromount Aqueous Mounting Medium (Sigma-Aldrich, MO, USA). The images were collected on Nikon Eclipse Ti-S microscope equipped with a Photometric CoolSNAP EZ turbo 1394 camera and analyzed on ImageJ (version 1.52t, NIH-Bethesda, MD, USA).

### RNA-sequencing and RT-qPCR

Total RNA was isolated from embryonic cardiac tissue using the Total RNA Purification kit (Norgen Biotek Corporation, ON, CA) according to the manufacturer’s instructions. For RNA-sequencing, the integrity and purity of the RNA were initially checked at the Bioanalyzer (Agilent 2100 bioanalyzer, Santa Clara, CA, USA) by using an RNA 6000 Nano Agilent kit. The RNA seq analysis was done at the EMBL service facility, Heidelberg. mRNA-Seq libraries were prepared from total RNA samples treated with DNase and single-end sequenced on the specified Illumina NextSeq 500 sequencing instrument. RNA-seq data are available online at the geo platform (GSE234392).

RNA-seq data (~37.7M reads per sample) were processed following the Illumina data processing pipeline. STAR aligner was used to map reads on the GRCm38-mm10 mouse reference genome. Subsequent data analysis was performed in R. Briefly, unexpressed genes were pre-filtered using a cut-off of 10 read counts. Count data of genes with non-zero expression (*n* = 24,697) were input into the DESeq2 package for differential expression analysis between *Pde2A*^*−/−*^ and *Pde2A*^*+/+*^ samples. The variance stabilizing transformation of data was done before PCA and hierarchical clustering of the heatmap. Functional enrichment analysis was performed using GSEA software and DAVID web tools.

cAMP and cGMP pathway analysis and comparison were performed using the annotations of the KEGG PATHWAY database.

For quantitative RT-PCR, mRNA was reverse transcribed to cDNA through Maxima H minus reverse transcriptase (Thermo Fischer Scientific, MA, USA). RT-qPCR reaction was carried out by using PowerUp SYBR Green Master Mix (Thermo Fisher Scientific, MA, USA). Target transcripts were analyzed using the Applied 7500 sequence detector system (Applied Biosystems, Carlsbad, CA, USA).

For the quantification analysis, the comparative threshold cycle (Ct) method was used. The Ct values of each gene were normalized to the Ct value of β-Act in the same RNA sample. The gene expression levels were evaluated by fold change using the equation 2^−ddCt^.

Primers used in mRNA analyses are reported in Supplementary Table S3.

### cAMP and cGMP assay

Embryonic hearts were homogenized and sonicated in 10 volumes of 0.1 M HCl with 0.1% Triton X-100 and processed with the Direct cAMP/cGMP ELISA kit (Enzo Life Science Inc., NY, USA) following the manufacturer’s instruction. Heart samples and standards were acetylated before being loaded onto the plate by using Acetic Anhydride and Triethylamine 1:2. Samples were incubated overnight at 4 °C on a plate shaker. A substrate solution was added and incubated for 1 hour at room temperature. The reaction was stopped, and samples were read by the spectrophotometer at an optical density of 405 nm. Results are referred to as cAMP and cGMP standard curves performed together with the cAMP and cGMP Assay.

### Reactive oxidative stress detection

Oxidative stress was quantified by measuring total reactive oxygen species (ROS) using 2 μM 2’,7’-dichlorodihydrofluorescein diacetate (CM-H2DCFDA; Thermo Fisher, MO, USA) staining and FACS analysis. Briefly, heart embryos, from untreated or NAC or Metaprolol treated mice, were dissected, washed in PBS, and dissociated with 5 mg/ml Collagenase Type II (Sigma-Aldrich, MO, USA) at 37 °C for 30 min. After washing with PBS, cells were resuspended in PBS and stained with CM-H2DCFDA for 30 min at 37 °C in the dark. At the end of the incubation, to remove the dye, cells were centrifuged and washed with PBS. The final cell suspensions were filtered through a 70 μm strainer, stained with Sytox Blue Dead Cell Stain (Thermo Fisher Scientific, MA, USA), and analyzed on a Cytoflex (Beckman Coulter, CA, USA). Flow cytometry data were analyzed with CytExpert software.

### Statistical analysis

All data are expressed as mean ± SEM and analyzed with Student’s *t*-test with two tails or ANOVA-one way or ANOVA-two way with Tukey correction. Differences were considered significant if *P < 0.05.

### Supplementary information

Supplementary Figure S1

Supplementary Figure S2

Supplementary Figure S3

Supplementary Figure S4

Supplementary Figure S5

Supplementary Figure S6

Supplementary table S1

Supplementary Table S2

Supplementary Table S3

Supplemental Video S1

Supplemental Video S2

Supplemental Video S3

Supplemental Video S4

Supplemental Video S5

Supplemental Video S6

Supplemental Video S7

Supplemental Video S8

Supplemental Video S9

aj-checklist final.pdf

## Data Availability

The data supporting this study are presented in the paper and in the supplementary information. Other information is available upon request. RNAseq data have been deposited in the Gene Expression Omnibus and are accessible through the accession number GSE234392.
