# Supplementary material for: Modulation of cAMP/cGMP signaling as prevention of congenital heart defects in Pde2A deficient embryos: a matter of oxidative stress
Source: Cell Death Dis. 2024 Feb 23;15(2):169. doi: 10.1038/s41419-024-06549-1 (PMC10891154; doi:10.1038/s41419-024-06549-1)
Supplement: Supplementary file 1 — Supplementary Figure S1 [file 41419_2024_6549_MOESM1_ESM.pdf]

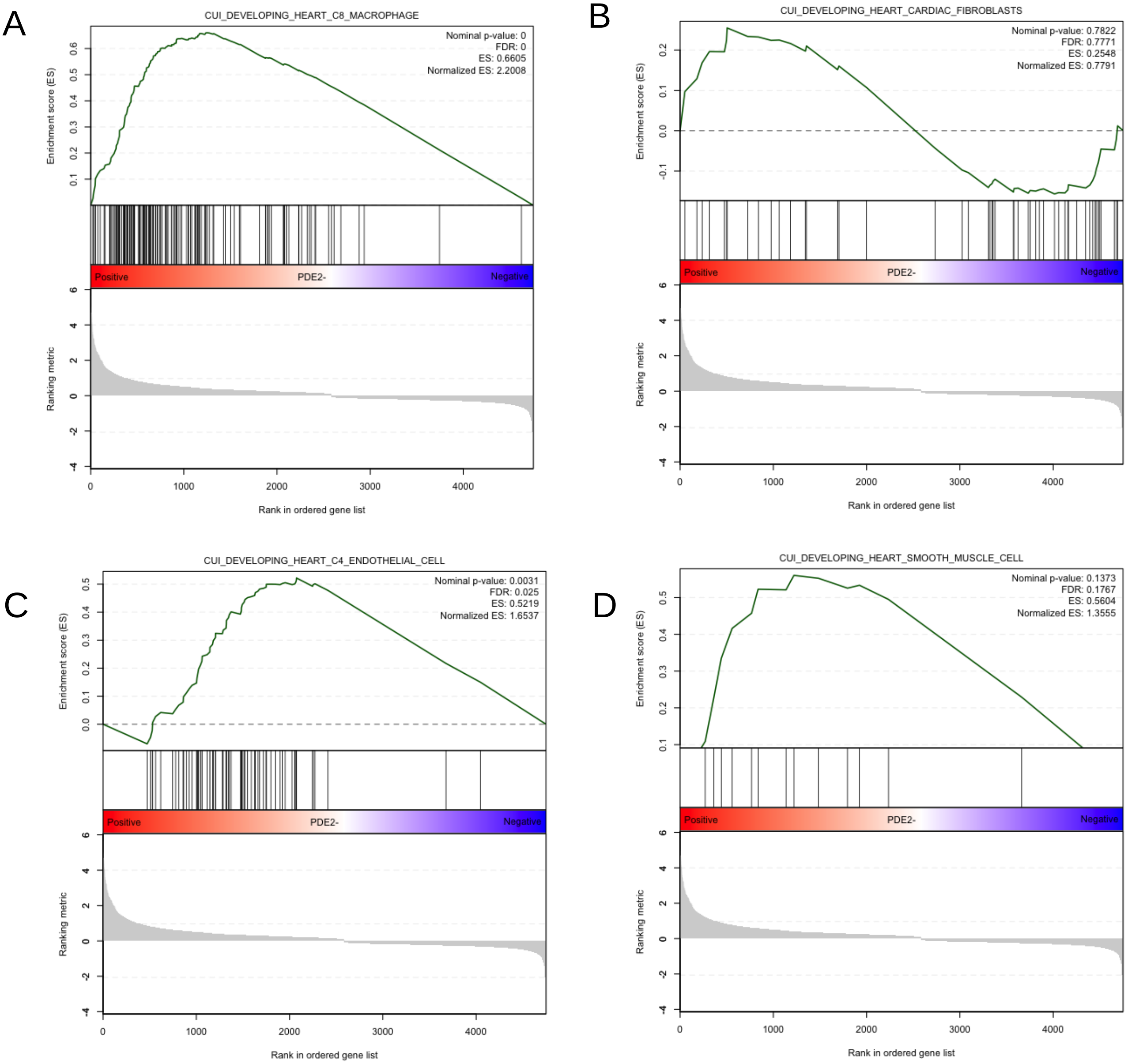

Fig. S1: Gene Set Enrichment Analysis (GSEA) of 515 *Pde2A*$^{-/-}$ differentially expressed genes by the gene sets from Molecular Signatures Database (MSigDB) for macrophage (A), fibroblast (B), endothelial (C) and smooth muscle cells (D); n=6 *Pde2A*$^{+/+}$ and n=5 *Pde2A*$^{-/-}$ hearts.
