# Supplementary material for: Modulation of cAMP/cGMP signaling as prevention of congenital heart defects in Pde2A deficient embryos: a matter of oxidative stress
Source: Cell Death Dis. 2024 Feb 23;15(2):169. doi: 10.1038/s41419-024-06549-1 (PMC10891154; doi:10.1038/s41419-024-06549-1)
Supplement: Supplementary file 2 — Supplementary Figure S2 [file 41419_2024_6549_MOESM2_ESM.pdf]

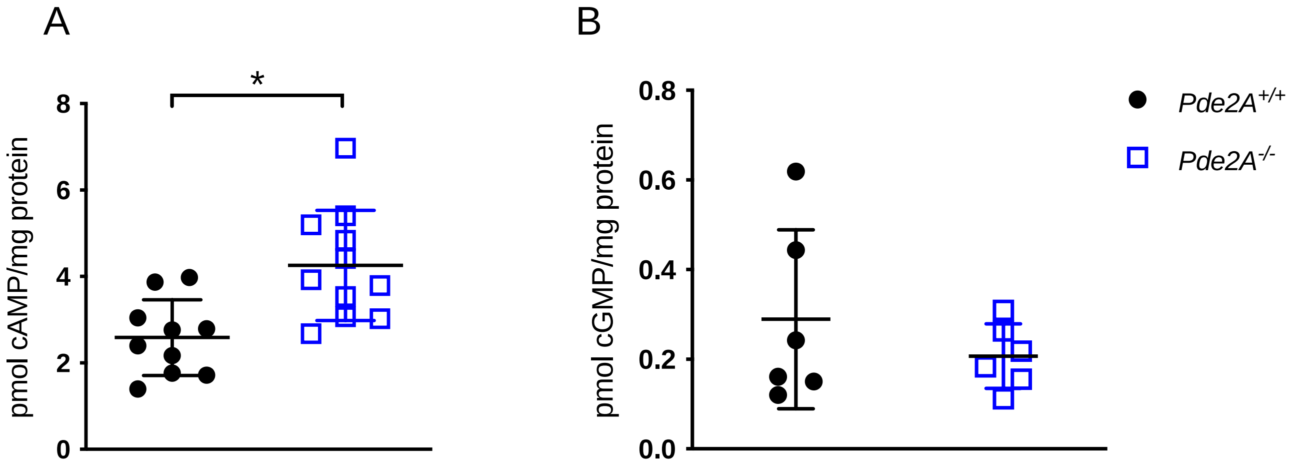

Fig. S2: cAMP but not cGMP levels are modified in hearts from *Pde2A*$^{-/-}$ embryos. A, B) cAMP and cGMP levels in *Pde2A*$^{+/+}$ and *Pde2A*$^{-/-}$ hearts. At least n=6 *Pde2A*$^{+/+}$ and n=7 *Pde2A*$^{-/-}$ hearts; unpaired Student's t-Test, * P≤0.05.
