# Supplementary material for: Modulation of cAMP/cGMP signaling as prevention of congenital heart defects in Pde2A deficient embryos: a matter of oxidative stress
Source: Cell Death Dis. 2024 Feb 23;15(2):169. doi: 10.1038/s41419-024-06549-1 (PMC10891154; doi:10.1038/s41419-024-06549-1)
Supplement: Supplementary file 3 — Supplementary Figure S3 [file 41419_2024_6549_MOESM3_ESM.pdf]

A

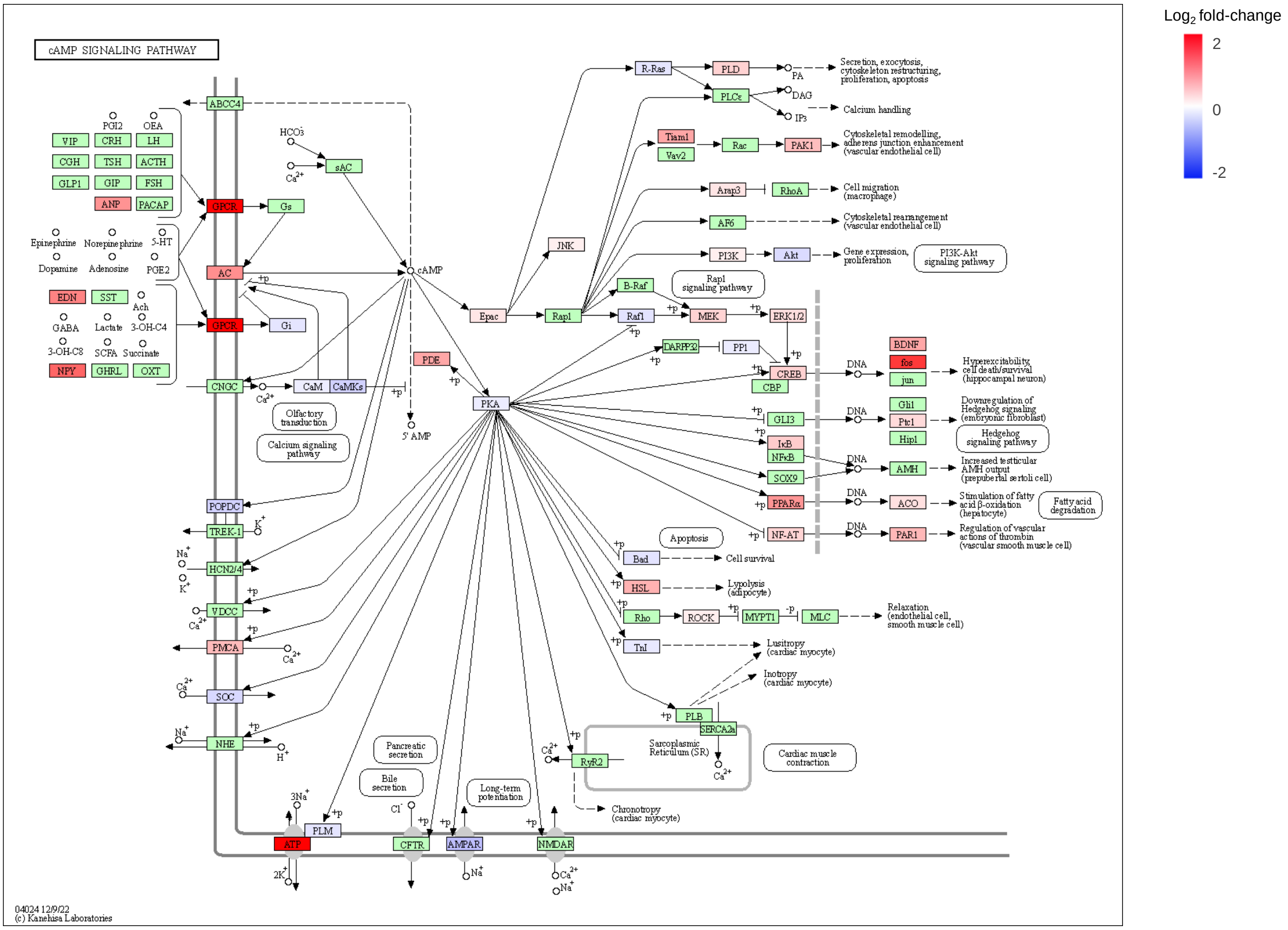

cAMP SIGNALING PATHWAY
ABCC4
PGI2
OEA
VIP
CRH
LH
CGH
TSH
ACTH
GLP1
GIP
FSH
ANP
PACAP
GPCR
Gs
HCO3-
sAC
Epinephrine
Norepinephrine
5-HT
Dopamine
Adenosine
PGE2
AC
cAMP
EDN
SST
Ach
GABA
Lactate
3-OH-C4
3-OH-C8
SCFA
Succinate
NPY
GHRL
OXT
GPCR
Gi
CNGC
CaM
CaMKs
Olfactory transduction
Calcium signaling pathway
PDE
5' AMP
POPDC
TREK-1
HCN2/4
VDCC
PMCA
SOC
NHE
ATP
PLM
CFTR
AMPAR
NMDAR
Pancreatic secretion
Bile secretion
Long-term potentiation
R-Ras
PLD
PA
DAG
IP3
PLCε
Secretion, exocytosis, cytoskeleton restructuring, proliferation, apoptosis
Calcium handling
Tiam1
Vav2
Rac
PAK1
Cytoskeletal remodelling, adherens junction enhancement (vascular endothelial cell)
Arap3
RhoA
Cell migration (macrophage)
AF6
Cytoskeletal rearrangement (vascular endothelial cell)
JNK
PI3K
Akt
Gene expression, proliferation
PI3K-Akt signaling pathway
B-Raf
Rap1 signaling pathway
Epac
Rap1
Raf1
MEK
ERK1/2
PKA
DARPP32
PP1
BDNF
fos
jun
CREB
CBP
DNA
Hyperexcitability, cell death/survival (hippocampal neuron)
GLI3
Gli1
Ptc1
Hip1
Downregulation of Hedgehog signaling (embryonic fibroblast)
Hedgehog signaling pathway
IκB
NFκB
SOX9
AMH
Increased testicular AMH output (prepubertal sertoli cell)
PPARα
ACO
Stimulation of fatty acid β-oxidation (hepatocyte)
Fatty acid degradation
NF-AT
PAR1
Regulation of vascular actions of thrombin (vascular smooth muscle cell)
Apoptosis
Bad
Cell survival
HSL
Lypolysis (adipocyte)
Rho
ROCK
MYPT1
MLC
Relaxation (endothelial cell, smooth muscle cell)
TnI
Lusitropy (cardiac myocyte)
Inotropy (cardiac myocyte)
PLB
SERCA2a
Sarcoplasmic Reticulum (SR)
Cardiac muscle contraction
RyR2
Chronotropy (cardiac myocyte)
04024 12/9/22
(c) Kanehisa Laboratories
Log2 fold-change
2
0
-2

B

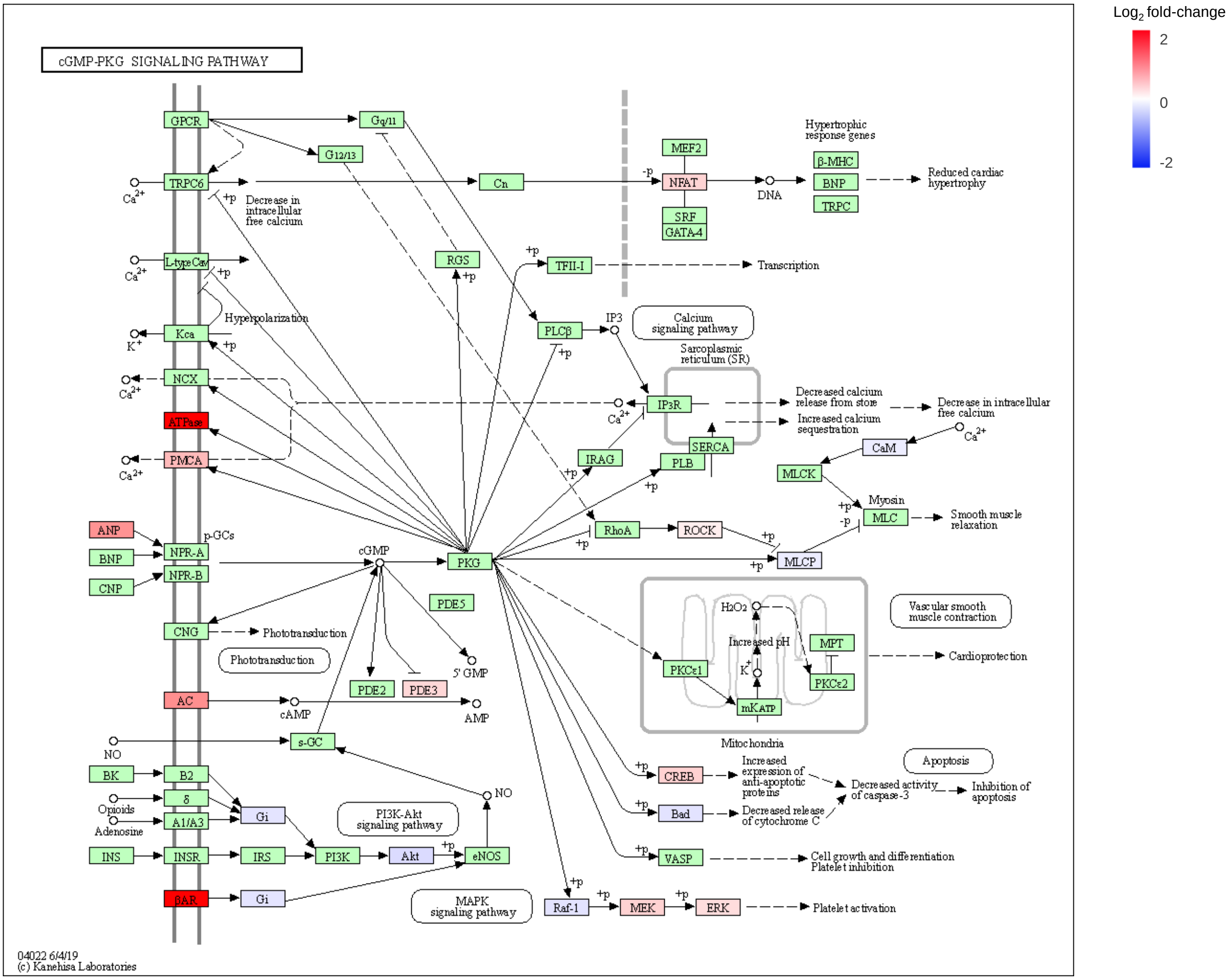

cGMP-PKG SIGNALING PATHWAY
GPCR
Gq/11
G12/13
TRPC6
Cn
Decrease in intracellular free calcium
L-type Cav
RGS
TFII-I
Transcription
Hyperpolarization
Kca
NCX
ATPase
PMCA
MEF2
NFAT
SRF
GATA-4
DNA
Hypertrophic response genes
β-MHC
BNP
TRPC
Reduced cardiac hypertrophy
PLCβ
IP3
Calcium signaling pathway
Sarcoplasmic reticulum (SR)
IP3R
Decreased calcium release from store
Increased calcium sequestration
Decrease in intracellular free calcium
CaM
MLCK
IRAG
PLB
SERCA
Myosin
MLC
Smooth muscle relaxation
RhoA
ROCK
MLCP
ANP
BNP
CNP
NPR-A
NPR-B
p-GCs
cGMP
PKG
PDE5
CNG
Phototransduction
PDE2
PDE3
5' GMP
AC
cAMP
AMP
NO
s-GC
BK
B2
Opioids
δ
Adenosine
A1/A3
Gi
PI3K-Akt signaling pathway
INS
INSR
IRS
PI3K
Akt
eNOS
βAR
MAPK signaling pathway
Raf-1
MEK
ERK
Platelet activation
H2O2
Increased pH
K+
MPT
PKCε1
PKCε2
mKATP
Mitochondria
Vascular smooth muscle contraction
Cardioprotection
Apoptosis
CREB
Increased expression of anti-apoptotic proteins
Decreased activity of caspase-3
Inhibition of apoptosis
Bad
Decreased release of cytochrome C
VASP
Cell growth and differentiation
Platelet inhibition
04022 6/4/19
(c) Kanehisa Laboratories
Log2 fold-change
2
0
-2

C

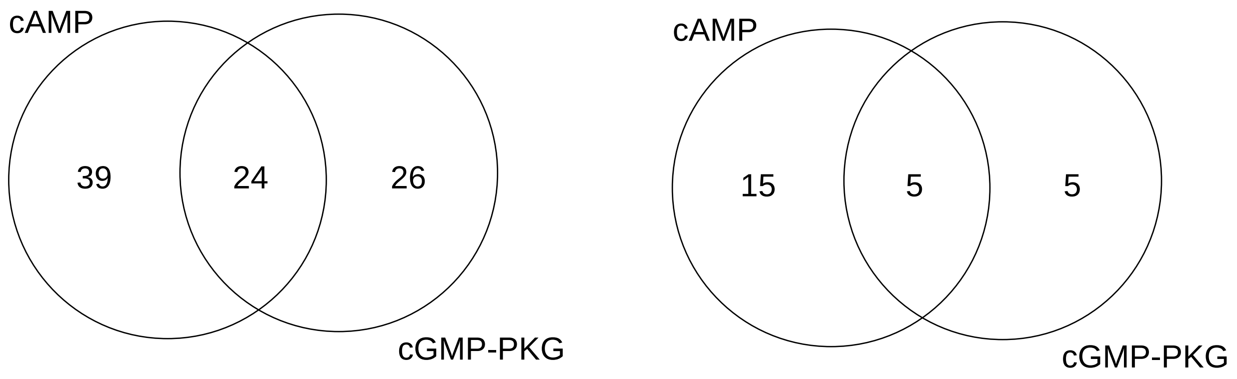

cAMP-specific	cAMP-cGMP	cGMP-specific
Adora2a	Adrb2	Adra2b
Bdnf	Adcy7	Bdkrb2
Camk4	Atp1a4	Irs2
Crhr2	Atp1b4	Mylk4
Edn1	Nppa	Nppb
Fos		
Gipr		
Glp1r		
Lipe		
Npy		
Npy1r		
Pde10a		
Pde4b		
Ppara		
Tiam1		

Fig. S3: Maps of cAMP (A) and cGMP-PKG (B) pathways by KEGG database and comparison of genes modulated in the two pathways (C). Up-regulated and down-regulated genes (p-adjusted < 0.05) in hearts of *Pde2A*$^{-/-}$ are indicated in red and blue, respectively (A, B, C). In (C) the Venn Diagrams show the common and specific modulated genes involved in cAMP and cGMP-PKG pathways (on the left), and among these, the highly modulated genes with absolute fold-change > 1.5 (on the right).
