# Supplementary material for: Modulation of cAMP/cGMP signaling as prevention of congenital heart defects in Pde2A deficient embryos: a matter of oxidative stress
Source: Cell Death Dis. 2024 Feb 23;15(2):169. doi: 10.1038/s41419-024-06549-1 (PMC10891154; doi:10.1038/s41419-024-06549-1)
Supplement: Supplementary file 4 — Supplementary Figure S4 [file 41419_2024_6549_MOESM4_ESM.pdf]

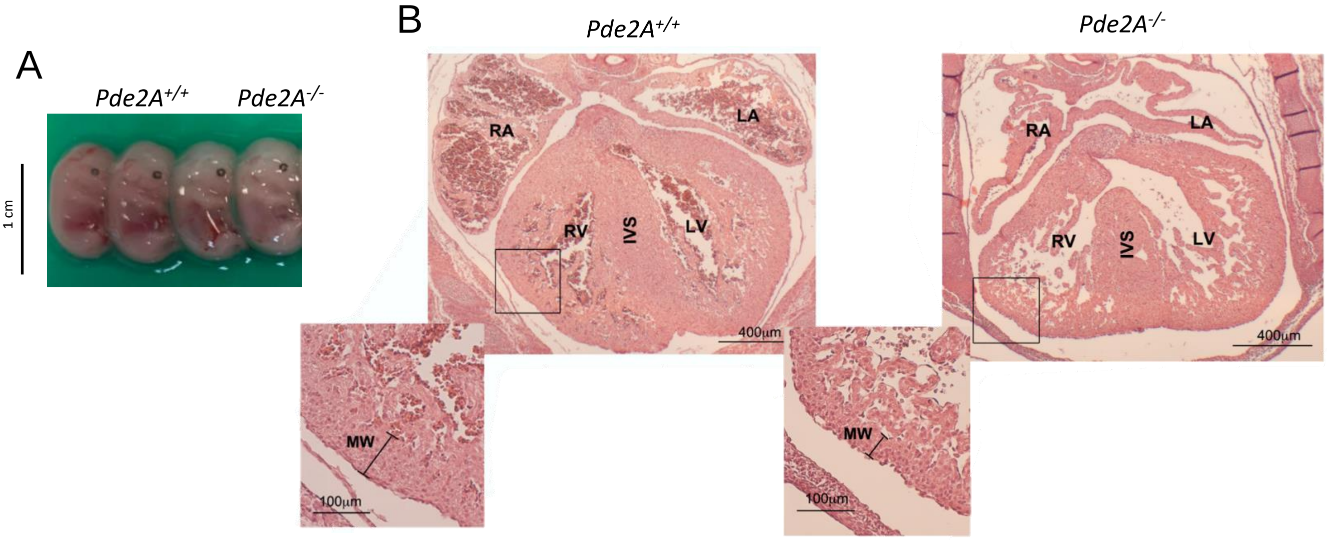

Fig. S4: A) Picture of *Pde2A*$^{+/+}$ and *Pde2A*$^{-/-}$ embryos at E14.5 Scale bar= 1 cm. B) Haematoxylin and Eosin staining of transversal sections of *Pde2A*$^{+/+}$ and *Pde2A*$^{-/-}$ embryos, the heart is shown. Left and right ventricles (LV, RV), atria (LA, RA) and interventricular septum (IVS) are indicated. Inset shows magnification of trabeculae and myocardial wall (MW).
