# Supplementary material for: Modulation of cAMP/cGMP signaling as prevention of congenital heart defects in Pde2A deficient embryos: a matter of oxidative stress
Source: Cell Death Dis. 2024 Feb 23;15(2):169. doi: 10.1038/s41419-024-06549-1 (PMC10891154; doi:10.1038/s41419-024-06549-1)
Supplement: Supplementary file 5 — Supplementary Figure S5 [file 41419_2024_6549_MOESM5_ESM.pdf]

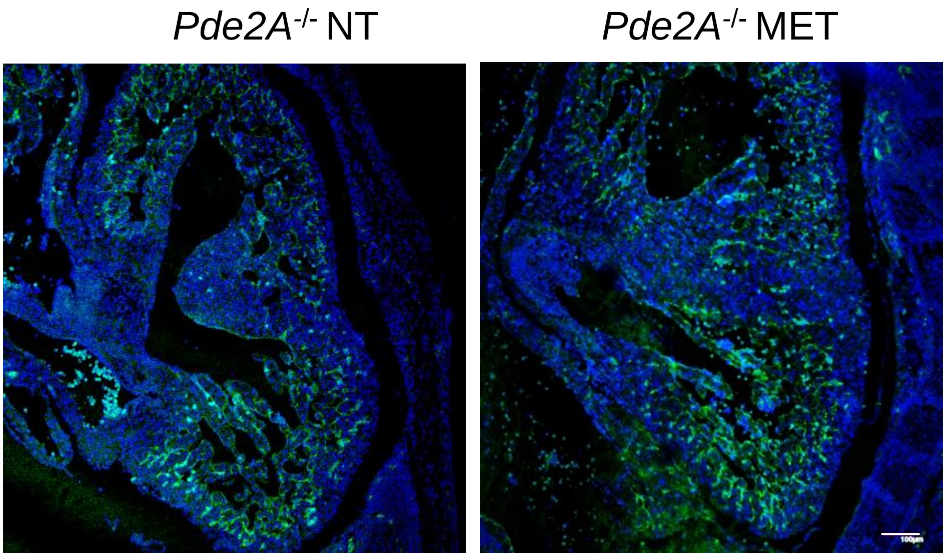


**Fig. S5:** Immunofluorescence with Endomucin (green) in E14.5 embryos detecting the endocardium network, n=2 each genotype and treatment. NT=not treated and MET=Metoprolol treated embryos. Hoechst was used to counterstain nuclei.
