# Supplementary material for: Modulation of cAMP/cGMP signaling as prevention of congenital heart defects in Pde2A deficient embryos: a matter of oxidative stress
Source: Cell Death Dis. 2024 Feb 23;15(2):169. doi: 10.1038/s41419-024-06549-1 (PMC10891154; doi:10.1038/s41419-024-06549-1)
Supplement: Supplementary file 6 — Supplementary Figure S6 [file 41419_2024_6549_MOESM6_ESM.pdf]

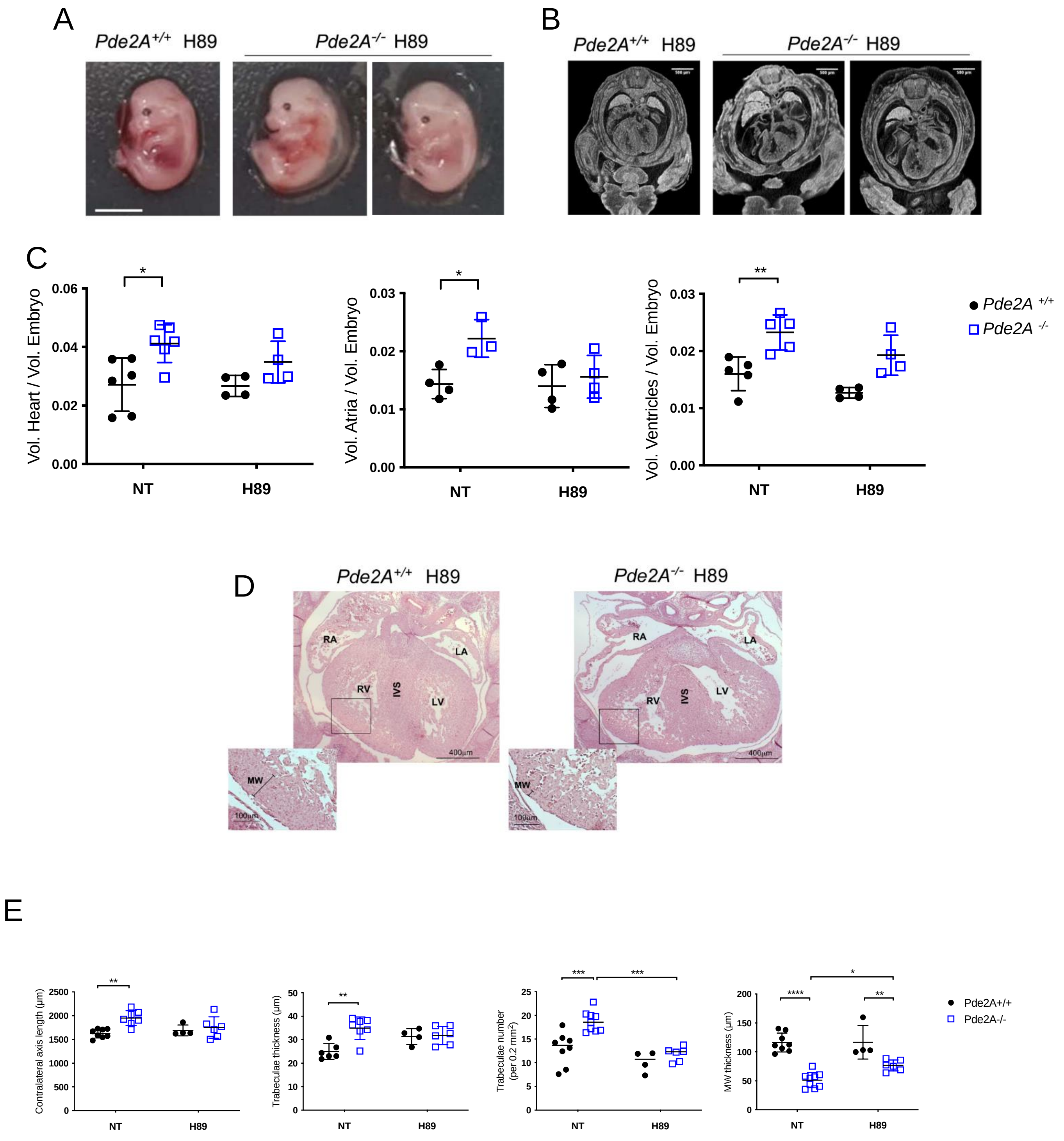

Fig. S6: Micro-CT and H&E examination show heart defects recovery after H89 treatment. A) Picture of *Pde2A*$^{+/+}$ and *Pde2A*$^{-/-}$ embryos at E14.5 treated with H89. Scale bar=0.5cm. B) Micro-CT picture of the hearts in H89 treated *Pde2A*$^{+/+}$ and *Pde2A*$^{-/-}$ embryos. C) Ratio between total heart, atrial, ventricular volumes relative to embryo volumes obtained by micro-CT analyses of H89 treated or not treated (NT) *Pde2A*$^{+/+}$ and *Pde2A*$^{-/-}$ embryos. At least n=4 for each genotype/treatment. D) Haematoxylin and Eosin staining of transversal sections of *Pde2A*$^{+/+}$ and *Pde2A*$^{-/-}$ H89 treated embryos, the heart is shown. Left and right ventricles (LV, RV), atria (LA, RA) and interventricular septum (IVS) are indicated. Inset shows magnification of trabeculae and myocardial wall (MW). E) Graphs of contralateral axis, trabeculae thickness and trabeculae number and myocardial analyses. At least =4 for each condition and genotype. ANOVA two-way was used to compare *Pde2A*$^{-/-}$ versus the relative *Pde2A*$^{+/+}$ in both conditions and *Pde2A*$^{-/-}$ not treated versus *Pde2A*$^{-/-}$ H89 samples. * $P \leq 0.05$, ** $P \leq 0.01$, *** $P \leq 0.001$.
