# Supplementary material for: Modulation of cAMP/cGMP signaling as prevention of congenital heart defects in Pde2A deficient embryos: a matter of oxidative stress
Source: Cell Death Dis. 2024 Feb 23;15(2):169. doi: 10.1038/s41419-024-06549-1 (PMC10891154; doi:10.1038/s41419-024-06549-1)
Supplement: Supplementary file 7 — Supplementary table S1 [file 41419_2024_6549_MOESM7_ESM.docx]

Supplementary Table S1: Knockout mouse models generated for the different Phosphodiesterases

| ***Pde isoform Knockouts*** | ***References*** | ***DOI*** |
| --- | --- | --- |
| **Pde1a** | Wang et al., 2017 | [10.1371/journal.pone.0181087](https://doi.org/10.1371%2Fjournal.pone.0181087) |
| **Pde1b** | Reed et al., 2002 | [10.1523/JNEUROSCI.22-12-05188.2002](https://doi.org/10.1523/jneurosci.22-12-05188.2002) |
| **Pde1c** | Cignar & Zhao, 2009 | [org/10.1038/nn.2289](https://doi.org/10.1038/nn.2289) |
| **Pde2a** | Stephenson et al., 2009 | [10.1369/jhc.2009.953471](https://doi.org/10.1369/jhc.2009.953471) |
| **Pde3a** | Masciarelli et al., 2004 | [10.1172/JCI21804](https://doi.org/10.1172%2FJCI21804) |
| **Pde3b** | Choi et al., 2006 | [10.1172/JCI24867](https://doi.org/10.1172/jci24867) |
| **Pde4a** | Hansen et al., 2014 | [10.1007/s00213-014-3480-y](https://doi.org/10.1007/s00213-014-3480-y) |
| **Pde4b** | Jin & Conti, 2002 | [10.1073/pnas.122041599](https://doi.org/10.1073/pnas.122041599) |
| Pde4c | Generated by Taconic | [www.taconic.com](http://www.taconic.com) |
| **Pde4d** | Jin et al., 1999 | [10.1073/pnas.96.21.11998](https://doi.org/10.1073/pnas.96.21.11998) |
| **Pde5a** | Gui et al., 2022 | [10.1055/a-1962-1613](https://doi.org/10.1055/a-1962-1613) |
| Pde6b | Yeo et al, 2019 | [10.1167/iovs.18-25556](https://doi.org/10.1167/iovs.18-25556) |
| **Pde7a** | Yang et al., 2003 | [10.4049/jimmunol.171.12.6414](https://doi.org/10.4049/jimmunol.171.12.6414) |
| **Pde7b** | Chevalier et al., 2012 | [10.1183/09031936.00102610](https://doi.org/10.1183/09031936.00102610) |
| **Pde8a** | Shimizu-Albergine et al., 2012 | [10.1124/mol.111.076125](http://dx.doi.org/10.1124/mol.111.076125) |
| **Pde8b** | Shimizu-Albergine et al., 2012 | [10.1124/mol.111.076125](http://dx.doi.org/10.1124/mol.111.076125) |
| **Pde9a** | Lee et al., 2015 | [10.1038/nature14332](https://doi.org/10.1038/nature14332) |
| **Pde10a** | Sciuciak et al., 2006 | [10.1016/j.neuropharm.2006.01.012](https://doi.org/10.1016/j.neuropharm.2006.01.012) |
| Pde11 | Wayman et al., 2005 | [10.1038/sj.ijir.3901307](https://doi.org/10.1038/sj.ijir.3901307) |

In bold *Pdes* expressed in the heart
