# Supplementary material for: Modulation of cAMP/cGMP signaling as prevention of congenital heart defects in Pde2A deficient embryos: a matter of oxidative stress
Source: Cell Death Dis. 2024 Feb 23;15(2):169. doi: 10.1038/s41419-024-06549-1 (PMC10891154; doi:10.1038/s41419-024-06549-1)
Supplement: Supplementary file 9 — Supplementary Table S3 [file 41419_2024_6549_MOESM9_ESM.docx]

**Table S3.** Primers used in mRNA analyses.

| Gene | Forward Primer | Reverse Primer |
| --- | --- | --- |
| Bmp10 | 5'-TCCATGCCGTCTGCTAACAT-3' | 5'-AGCCATGACGACCTCTTCAT-3' |
| β-Act | 5'-TGACAGGATGCAGAAGA-3’ | 5'-GTACTTGCGCTCAGGAGGAG-3’ |
| Bnp | 5'-AAGCTGCTGGAGCTGATAAGA-3 | 5'-ATCCGATCCGGTCTATCTTG-3 |
| C3 | 5'-CGCAACGAACAGGTGGAGATC-3' | 5'-CTGGAAGTAGCGATTCTTGGCG-3' |
| Ccl12 | 5' GCTACAGGAGAATCACAAGCAGC3' | 5'-ACGTCTTATCCAAGTGGTTTATGG-3' |
| Ccl2 | 5'-AGGTCCCTGTCATGCTTCTG-3 | 5'-TCTCCAGCCTACTCATTGGG-3 |
| Cxcl1 | 5'-TCCAGAGCTTGAAGGTGTTGCC-3' | 5'-AACCAAGGGAGCTTCAGGGTCA 3' |
| Cxcl10 | 5'-AAGTGCTGCCGTCATTTTCT-3’ | 5'-CTTCCCTATGGCCCTCATTC-3’ |
| Cxcl16 | 5'-TCCTTTTCTTGTTGGCGCTG-3’ | 5'-CAGCGACACTGCCCCTGGT-3’ |
| Epor | 5'-TAGGCCCCCTCTGTCTCCTAC-3' | 5'-CAAGTCTTCCAAGCGTTGGGT-3' |
| Fgf23 | 5'-GGACCAGCTATCACCTACAGA-3' | 5'-TCATGGCTCCTGTTATCACC-3' |
| Gata1 | 5'-CTAAGGTGGCTGAATCCTCTGC-3' | 5'-CCTGTGGAATCTGATGGTGAGG 3' |
| Gpx1 | 5'-CGCTCTTTACCTTCCTGCGGAA-3' | 5'-AGTTCCAGGCAATGTCGTTGCG-3' |
| Hif | 5-GGCGAGAACGAGAAGAAAAA-3 | 5'-GAGCTCACATTGTGGGGAAG-3’ |
| Hspa1a | 5'-ACAAGTCGGAGAACGTGCAGGA-3' | 5'-GTTGTCCGAGTAGGTGGTGAAG-3' |
| Il1a | 5'-ACGGCTGAGTTTCAGTGAGACC-3' | 5'-CACTCTGGTAGGTGTAAGGTGC-3' |
| Irf7 | 5'-CCTCTGCTTTCTAGTGATGCCG-3' | 5'-CGTAAACACGGTCTTGCTCCTG-3' |
| Klf1 | 5'-CGGCGAACTTTGGCACCTAAGA-3' | 5'-AGGAGCAGGCATAAGGCTTCTC-3' |
| Ncf1 | 5'-GCTGACTACGAGAGGAGTTCGG-3' | 5'-CCTCGCTTTGTCTTCATCTGGC-3' |
| Ncf2 | 5'-GCAGAAGAGCAGTTGGCATTGG-3' | 5'-CTGCCTCTCATTTGGACGGAAC-3' |
| Nox2 | 5'-TGGCGATCTCAGCAAAAGGTGG-3' | 5'-GTACTGTCCCACCTCCATCTTG-3' |
| Nrf2 | 5'-CAGCGACGGAAAGAGTATGA-3’ | 5'-TGGGCAACCTGGGAGTAG-3’ |
| Serpin1 | 5'-TCATCAGACAATGGAAGGGC-3’ | 5'-GCCAGGGTTGCACTAAACAT-3’ |
| Stat1 | 5'-GCCTCTCATTGTCACCGAAGAAC-3' | 5'-TGGCTGACGTTGGAGATCACCA-3' |
| Stat3 | 5'-AGGAGTCTAACAACGGCAGCCT-3' | 5'-GTGGTACACCTCAGTCTCGAAG-3' |
| Ucp2 | 5'-TAAAGGTCCGCTTCCAGGCTCA-3' | 5'-ACGGGCAACATTGGGAGAAGTC-3' |
| Vegfa | 5'-GATCATGCGGATCAAACCTC-3’ | 5'-AATGCTTTCTCCGCTCTGAA 3’ |
